# Targeting pyruvate kinase M2 for the treatment of kidney disease

**DOI:** 10.3389/fphar.2024.1376252

**Published:** 2024-06-07

**Authors:** Dan-Qian Chen, Jin Han, Hui Liu, Kai Feng, Ping Li

**Affiliations:** ^1^ College of Life Sciences, Northwest University, Xi’an, Shaanxi, China; ^2^ Department of Nephrology, Xi’an Chang’an District Hospital, Xi’an, Shaanxi, China; ^3^ Beijing Key Lab for Immune-Mediated Inflammatory Diseases, Institute of Clinical Medical Sciences, China-Japan Friendship Hospital, Beijing, China

**Keywords:** pyruvate kinase M2, diabetic kidney disease, acute kidney injury, post-translational modification, glycolysis

## Abstract

Pyruvate kinase M2 (PKM2), a rate limiting enzyme in glycolysis, is a cellular regulator that has received extensive attention and regards as a metabolic regulator of cellular metabolism and energy. Kidney is a highly metabolically active organ, and glycolysis is the important energy resource for kidney. The accumulated evidences indicates that the enzymatic activity of PKM2 is disturbed in kidney disease progression and treatment, especially diabetic kidney disease and acute kidney injury. Modulating PKM2 post-translational modification determines its enzymatic activity and nuclear translocation that serves as an important interventional approach to regulate PKM2. Emerging evidences show that PKM2 and its post-translational modification participate in kidney disease progression and treatment through modulating metabolism regulation, podocyte injury, fibroblast activation and proliferation, macrophage polarization, and T cell regulation. Interestingly, PKM2 activators (TEPP-46, DASA-58, mitapivat, and TP-1454) and PKM2 inhibitors (shikonin, alkannin, compound 3k and compound 3h) have exhibited potential therapeutic property in kidney disease, which indicates the pleiotropic effects of PKM2 in kidney. In the future, the deep investigation of PKM2 pleiotropic effects in kidney is urgently needed to determine the therapeutic effect of PKM2 activator/inhibitor to benefit patients. The information in this review highlights that PKM2 functions as a potential biomarker and therapeutic target for kidney diseases.

## 1 Introduction

Kidney is a highly metabolically active organ. The metabolic programing in the kidney is different in the distinct regions of kidney. The cortex has high rates of gluconeogenesis and fatty acid oxidation, while the medulla mainly relies on glycolysis, which indicates that oxygen requirement reduces gradually from cortex to medulla in kidney. In the cortex, proximal tubules are highly aerobic and mainly responsible for fluid reabsorption, while in the medulla, distal nephron segments have lower oxygen tension. Under physiological condition, the medulla papilla and distal convoluted tubules has high glycolytic activity (Tang and Wei, 2023), and glycolysis is also the major energy source of podocytes (Brinkkoetter et al., 2019). Even proximal tubular cells have low glycolytic activity, glycolysis plays an important role in maintaining phosphate homeostasis (Zhou et al., 2022; Zhou et al., 2023). Since emerging evidences demonstrated that glycolysis is significantly disturbed during kidney disease (Nakagawa et al., 2020; Gu et al., 2022; Ito et al., 2022), glycolysis is the potential therapeutic target for kidney disease.

**FIGURE 1:**
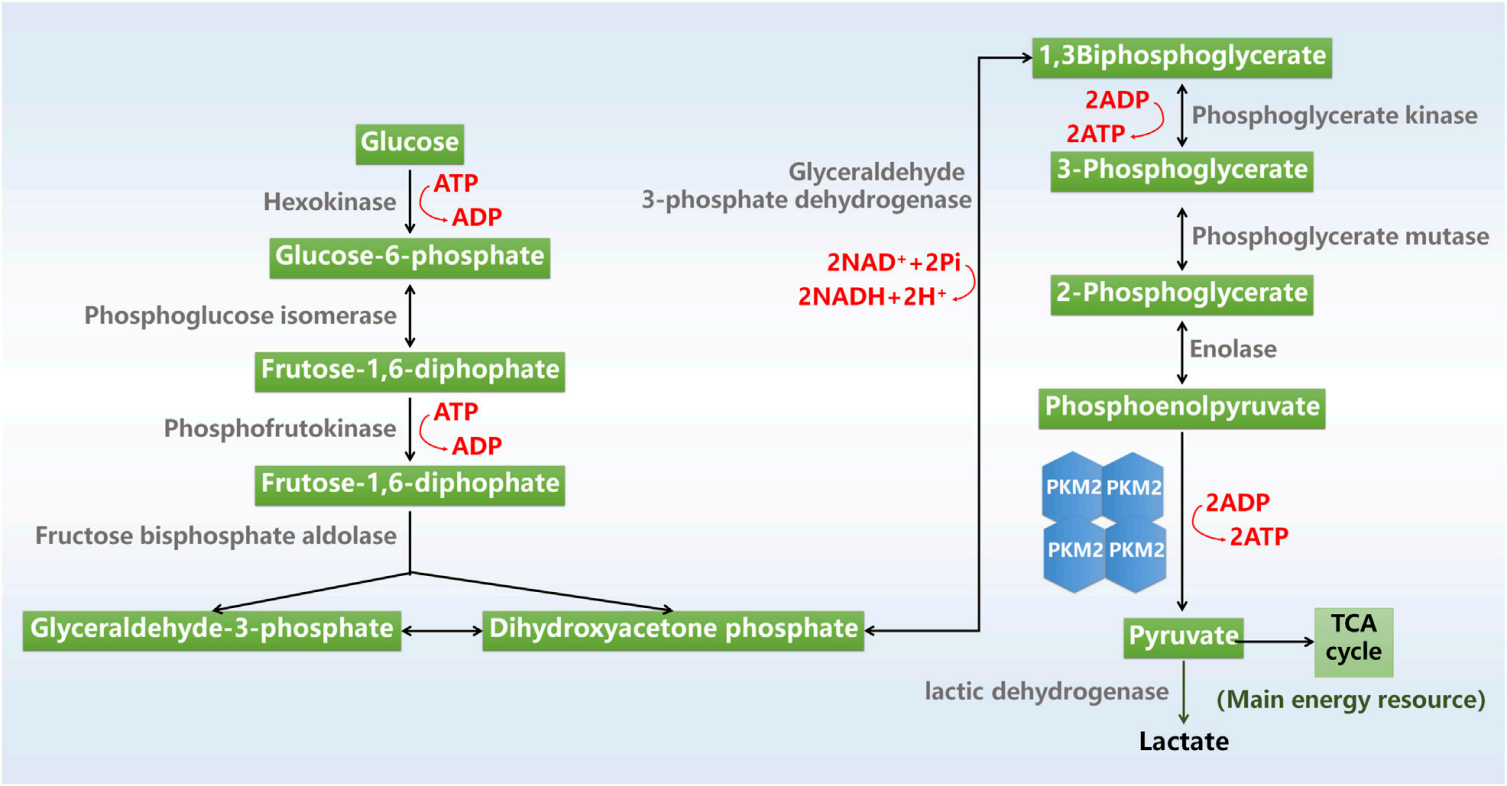
Glycolysis process. ADP, adenosine triphosphate; ATP, adenosine triphosphate; NAD, nicotinamide adenine dinucleotide; TCA cycle, tricarboxylic acid cycle.

Glycolysis is a major metabolic process that converts glucose into pyruvate with the production of adenosine triphosphate (ATP) and nicotinamide adenine dinucleotide. Glycolysis is mediated by a series of cellular enzymes, and hexokinase, phosphofructokinase and pyruvate kinase (PK) serves as key rate-limiting enzymes. The final product of glycolysis pyruvate is converted to lactic acid or acetyl-coenzyme A (CoA) for utilization in tricarboxylic acid cycle by mitochondria, which produces energy precursors for oxidative phosphorylation (Figure 1).

PK mediates its substrate phosphoenolpyruvate (PEP) to pyruvate. PK has four different subtypes including L, R, M1, and M2 (Zhang et al., 2019). PKL isoforms are mainly distributed in liver, pancreas and kidney, and PKR is distributed in erythrocytes (Puckett et al., 2021). PKM1 are predominantly expressed in muscle, mature spermatozoa and central nervous system, while PKM2 is expressed in brain, kidney, lung and spleen (Alquraishi et al., 2019; Puckett et al., 2021). PKM2 is the dominant form of PK in kidney tissue (Alquraishi et al., 2019), and a lot of researches have revealed that regulating PKM2 affects kidney disease progression and treatment (Li et al., 2020a; Chen et al., 2023; Xie et al., 2023), highlighting the important role of PKM2 in kidney disease.

In this review, we describe some important research progresses of PKM2 in recent 5 years, from its structure and post-translational modifications to its role in kidney, and introduce the potential intervention effects of PKM2 agonists and antagonists. We further present clinical application of PKM2 in kidney disease, with the goal of highlighting the therapeutic potential of PKM2 in kidney disease. Finally, we discuss the major opportunities and obstacles of PKM2 in kidney disease to facilitate the clinical treatment.

## 2 PKM2 structure and its post-translational modifications

### 2.1 PKM2 structure

**FIGURE 2:**
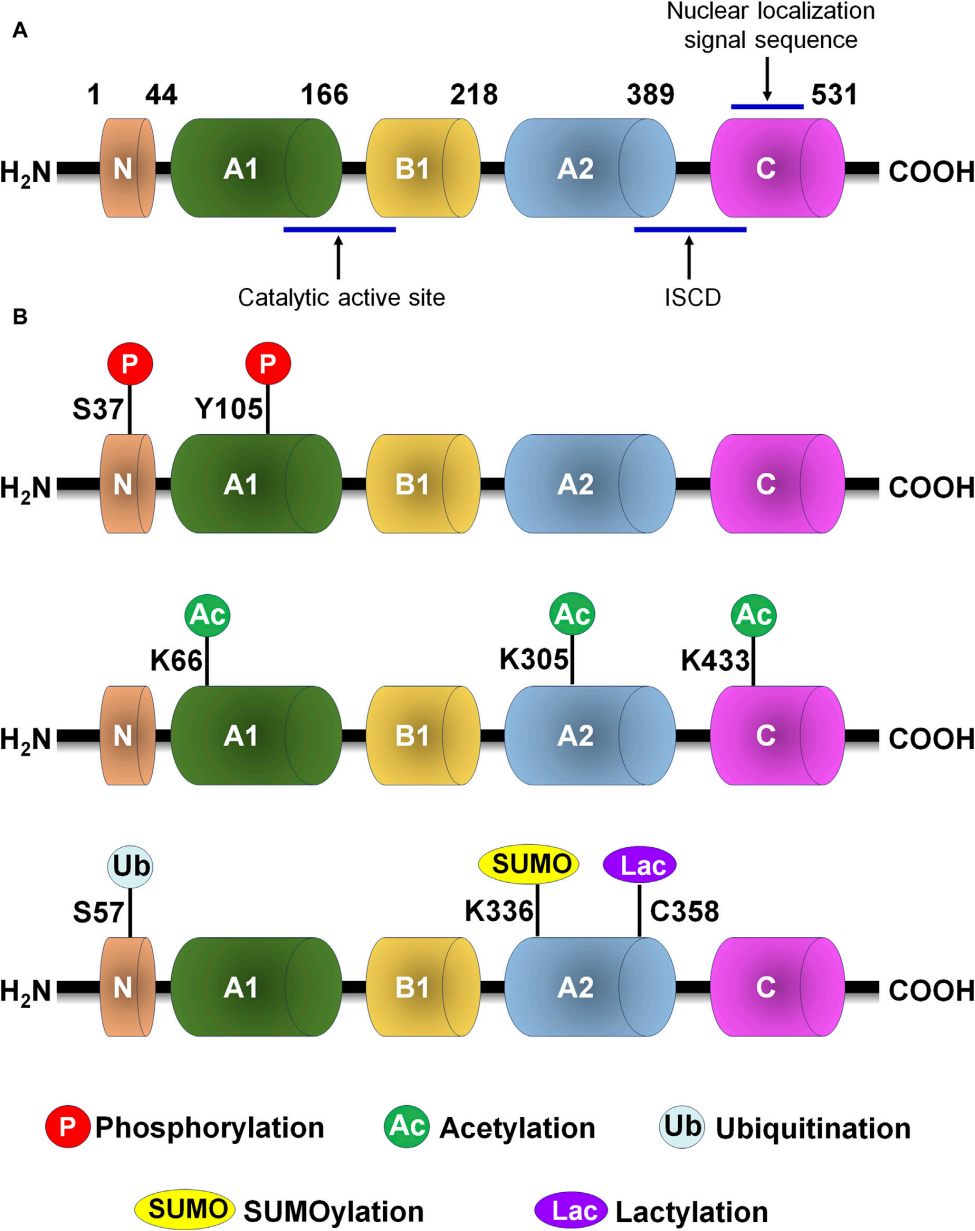
PKM2 protein structure and post-translational modification of PKM2. **(A)** PKM2 protein structure. **(B)** The major post-translational modification sites of PKM2, including phosphorylation, acetylation, ubiquitination, SUMOylation and lactylation. C, cysteine; ISCD, intersubunit contact domain; K, lysine; S, serine; Y, tyrosine.

Human PKM2 protein consists of 531 amino acids that is subdivided as N (43aa), A (244aa), B (102aa) and C (142aa) domains according to their characteristic features (Figure 2A). The catalytic active site locates at the adjoining region between the A1 and B domains, while the intersubunit contact domain (ISCD) locates at the adjoining region between the A2 and C domains. Nuclear localization signal sequence and the binding site of the allosteric activator (FBP) locate at C domain. The A domain mediates the subunits interaction to form a dimer and functions as the core of monomer, while the formation of the tetramer form of PKM2 is assembled through the binding of two dimers’ C-subunits in ISCD.

The enzyme activity of PKM2 is affected by its oligomeric state. PKM2 has four different enzymatic states: an inactive monomer, a nearly inactive dimer, an inactive T state tetramer, and an active R state tetramer (Prakasam et al., 2018; Alquraishi et al., 2019). The tetrameric form has a high PEP affinity, while the dimeric form has a low affinity for PEP and is almost inactive under physiological conditions.

### 2.2 PKM2 post-translational modification

Numerous conserved post-translational modification sites exist in PKM2 protein, including phosphorylation, acetylation, methylation, SUMOylation and oxidation (Figure 2B; Table 1). Post-translational modification of PKM2 determines its structural and functional properties, including oligomeric state, catalytic activity, protein stability, binding of allosteric activators, conditional protein interaction, and subcellular localization, which influences disease progression and therapeutic effect.

**TABLE 1:** Post-translational modifications of PKM2 and their effects in kidney and other disease.

Modification	Specific site	Effects	Disease	Proposed function	References
Phosphorylation	Tyr105	Increasing glycolysis	Autoimmune diseases	Modulating Th17 cell metabolic reprograming	Chen et al. (2022)
		Facilitating tetramer formation	Nonalcoholic steatohepatotis	Modulating macrophage polarization	Xu et al. (2020)
		Promoting dimer formation	AKI	Promoting mitochondrial fragmentation and suppresses renal tubular injury and cell death	Xie et al. (2023)
		Suppressing glycolysis	Osteoclast	Suppressing osteoclastic bone loss and modulating osteoclast differentiation	Kim et al. (2023)
	Ser37	Decreasing nuclear translocation	Breast cancer	Reducing cell invasion, impairing redox balance, and triggering cancer cell death	Apostolidi et al. (2021)
	—	Promoting nuclear translocation	Liver fibrosis	Enhancing glycolysis and M1 polarization	Rao et al. (2022)
Acetylation	Lys433	Promoting detetramerization and nuclear translocation	Aberrant immune responses	Regulating dendritic cell activation, promoting glycolysis and fatty acid synthesis	Jin et al. (2020), Wu et al. (2023)
		Promoting nuclear translocation	Lung cancer	Promoting cell migration	Biyik-Sit et al. (2021)
		Promoting glycolysis	Innate immune cell-mediated inflammation	Inhibiting inflammatory effect	Das Gupta et al. (2020)
		Stabilizing Bcl-2	Lung ischemia/reperfusion injury	Increasing apoptosis	Zhao et al. (2022b)
	Lys305	Suppressing PKM2 nuclear translocation	Heaptocellular carcinoma	Activating dendritic cell, and facilitating glycolysis and fatty acid synthesis	Wu et al. (2023)
	Lys66	Increasing PKM2 expression	Hematologic diseases	Promoting hematopoietic imbalance	Zhang et al. (2022)
SUMOylation	—	Promoting PKM2 phosphorylation and nuclear translocation and reducing enzymatic activity	Rheumatoid arthritis	Reducing glycolysis, aggressive phenotype, and inflammation	Wang et al. (2020)
	—	Increasing Ectosomal PKM2 excretion	Heaptocellular carcinoma	Inducing monocyte-to-macrophage differentiation and tumor microenvironment remodeling	Hou et al. (2020)
	Lys270	Promoting dimeric formation and and nuclear translocation	Leukemia	Promoting the blockage of myeloid differentiation	Xia et al. (2021)
	Lys336	Increasing glycolysis	Lung cancer	Enhancing glycolysis and cell proliferation	An et al. (2018)
	—	Increasing glycolysis	Heaptocellular carcinoma	Promoting glycolytic reprogramming, growth and metastasis	Zhou et al. (2022a)
Ubiquitination	—	Facilitating degradation	Colorectal cancer	Modulating growth and metastasis	Zhao et al. (2022a)
	—		Intrahepatic cholangiocarcinoma	Suppressing tumor cell migration, invasion, and proliferation	Chen et al. (2021)
Deubiquitination	Ser57	Preventing degradation	—	Functioning as a HAUSP binding substrate	Choi et al. (2020)
	—	Increasing enzymatic activity	Colorectal cancer	Increasing glucose consumption, lactate production, and cellular ATP production	Yu et al. (2022)
Lactylation	Lys62	Promoting dimer formation and inhibiting nuclear translocation	Metabolic adaptation	Modulating metabolic adaptions in pro-inflammatory macrophages	Wang et al. (2022a)
O-GlcNAcylation	—	Suppressing enzymatic activity	Tumor cells	Promoting aerobic glycolysis and tumor growth	Singh et al. (2020)
S-nitrosylation	—	Suppressing enzymatic activity	AKI	Aggravating kidney injury	Zhou et al. (2023a)
Modification by acrolein	Cys358	Suppressing enzymatic activity	DKD	Facilitating epithelial-mesenchymal transition	Kuo et al. (2023)

AKI, acute kidney injury; DKD, diabetic kidney disease.

The Tyr105 and Ser37 are the common phosphorylation site of PKM2 phosphorylation, which mainly control PKM2 active tetramer formation and nuclear translocation. CoA binds to PKM2 to block its Tyr105 phosphorylation and nuclear translocation, resulting in reduced glycolysis in Th17 cell. These data indicate that targeting Th17 cell metabolic reprograming via PKM2 functions as a potential therapeutic intervention for Th17 cell-associated autoimmune diseases (Chen et al., 2022). Annexin A5 has the ability to switch metabolic reprogramming from glycolysis to oxidative phosphorylation in activated macrophages by modulating PKM2 post-translational modification. Mechanistically, Annexin A5 directly interacts with PKM2 and further reduces Tyr105 phosphorylation to facilitate tetramer formation in macrophage polarization (Xu et al., 2020). The phosphorylation of PKM2 at Tyr105 promotes its dimer formation and translocation into the mitochondria after treatment with staurosporine or cisplatin in acute kidney injury (AKI). Mitochondrial PKM2 binds myosin heavy chain 9 to facilitate dynamin-related protein 1-mediated mitochondrial fragmentation, and the loss of PKM2 attenuates mitochondrial fragmentation and suppresses renal tubular injury and cell death (Xie et al., 2023). Immunoglobulin superfamily 11 suppresses PKM2 activity through promoting PKM2 phosphorylation at Tyr105 to modulate osteoclast differentiation. Modulating PKM2 activity is considered as a metabolic switch that is necessary for optimal osteoclast maturation (Kim et al., 2023). The phosphorylation of PKM2 at Tyr105 and Ser37 can be activated by an allosteric activator, TEPP-46, results in PKM2 tetramerization and inhibits its nuclear translocation to prevent glycolysis, which reduces CD4^+^ T cell pathogenicity and inhibits autoimmunity (Angiari et al., 2020). PKM2 phosphorylation involves in aggressive breast cancer cell phenotypes, and PKM2 phosphorylation at Ser37 functions as an effective therapeutic target for triple-negative breast cancer treatment (Apostolidi et al., 2021). Follistatin-like protein 1 (FSTL1), a secreted glycoprotein, could directly bound to PKM2 through its FK domain. FSTL1 facilitates PKM2 phosphorylation and nuclear translocation and inhibits PKM2 ubiquitination to enhance PKM2-dependent glycolysis and M1 polarization to promote liver fibrosis (Rao et al., 2022). In addition, succinate stimulates PKM2 dimerization that further translocate into the nucleus and mitochondria to accelerate fibroblast activation and apoptosis resistance in heart (Wang et al., 2023).

The acetylation of PKM2 occurs in Lys433, Lys305, and Lys66, and this process can be reversed by the deacetylase. JNK induces the acetylation of PKM2 at Lys433, resulting in PKM2 detetramerization and nuclear translocation during dendritic cell activation through modulating glycolysis and fatty acid synthesis (Jin et al., 2020). Testes-specific protease 50 (TSP50) maintains the low activity of PKM2 to control aerobic glycolysis in heaptocellular carcinoma (HCC) cells. Mechanistically, TSP50 enhances the acetylation of PKM2 at Lys433, while the acetyl-insensitive mutation of PKM2 K433R obviously counteracts TSP50-induced aerobic glycolysis (Gao et al., 2021). Phosphoserine aminotransferase 1 (PSAT1) accumulates nuclear PKM2 translocation to facilitate lung cancer cell migration, while the acetyl-mimetic mutant of PKM2 (K433Q) affected PSAT1-mediated cell migration (Biyik-Sit et al., 2021). Class IIa histone deacetylase HDAC7 mediates the deacetylation of PKM2 at Lys433 to aggravate its proinflammatory property (Das Gupta et al., 2020). Sirt3 mediates the deacetylation of PKM2 at Lys433 in A549 cells, which process significantly alleviates apoptosis against lung ischemia/reperfusion injury (Zhao et al., 2022). Phosphoglycerate dehydrogenase (PHGDH) increases the stability and activity of PKM2 through interacting with PKM2 to facilitate Lys433 acetylation and prevent Lys305 acetylation, which leading to PKM2 nuclear translocation and ultimately prevents premature senescence (Wu et al., 2023). A common environmental carcinogen, 1,4-benzoquinone, results in the acetylation of PKM2 at Lys66 that contributes to the upregulation of PKM2. However, the acetyltransferase general control non-derepressible 5 could reverse the acetylation of PKM2 at Lys66 (Zhang et al., 2022). Besides, Sirt2 mediates PKM2 deacetylation, which is the potential therapeutic approach for psoriasis therapy (Hao et al., 2021).

The SUMOylation often occurs in lysine residue in PKM2. Small ubiquitin-like modifier-activating (SUMO-activating) enzyme 1/ubiquitin like modifier activating enzyme 2 mediates the SUMOylation of PKM2 that facilitates PKM2 phosphorylation and nuclear translocation and reduces enzymatic activity (Wang et al., 2020). HCC-derived ectosomal PKM2 induced metabolic reprogramming in monocytes to accelerate HCC progression. In HCC cells, the SUMOylation of PKM2 facilitates its plasma membrane targeting and subsequent ectosomal excretion. Ectosomal PKM2 is clearly detected in the plasma of HCC patients and functions as a potential diagnostic marker for HCC (Hou et al., 2020). The SUMOylation of PKM2 at Lys270 induces PKM2 from tetrameric to dimeric formation, and nuclear translocation. The replacement of wild type PKM2 to a SUMOylation-deficient mutant (K270R) weakens PKM2 effect on RUNX1 in leukemia cells (Xia et al., 2021). The knockdown of small ubiquitin-like modifier 1 (SUMO1) causes the downregulation of PKM2 protein in A549 cells. SUMO1 directly mediates the SUMO1 modification of PKM2 at Lys336 that enhances glycolysis and cell proliferation (An et al., 2018). The activated guanosine triphosphate binding protein 4 (GTPBP4) promotes PKM2 SUMOylation via facilitating SUMO1 protein activation and functioning as a linker between SUMO1 and PKM2 protein, which process accelerates aerobic glycolysis in HCC (Zhou et al., 2022).

The ubiquitination and deubiquitination of PKM2 are reported in plenty of researches. DExD-box helicase 39B (DDX39B) directly interacts with PKM2 and stabilizes PKM2 by competitively suppressing STIP1 homology and U-box-containing protein 1 (STUB1)-mediated PKM2 ubiquitination and degradation. DDX39B accelerates the nuclear translocation of PKM2 to promote the activation of oncogenes and glycolysis-related genes, which process is independent of ERK1/2-mediated phosphorylation of PKM2 at Ser37 (Zhao et al., 2022). CNRIP1 overexpression activates Parkin (an E3 ubiquitin ligase), and triggers the protein degradation of PKM2 in intrahepatic cholangiocarcinoma cells (Chen et al., 2021). In addition, the deubiquitinating enzyme, herpesvirus-associated ubiquitin-specific protease (HAUSP), interacts with PKM2 at Ser57 to deubiquitinate PKM2, while HAUSP knockdown increases PKM2 ubiquitination (Choi et al., 2020). OTUB2, an OTU deubiquitinase, directly interacts with PKM2 and suppresses its ubiquitination via weakening the interaction of PKM2 and Parkin to increase PKM2 activity and glycolysis in colorectal cancer (Yu et al., 2022).

Additionally, the lactylation of PKM2 is found in macrophages, and Lys62 site is responsible for PKM2 lactylation to inhibit inflammatory metabolic adaptation. Lactate facilitates PKM2 lactylation that delays the transition from tetramer to dimer, and increase its enzymatic activity and suppress nuclear distribution (Wang et al., 2022). O-GlcNAc transferase mediates PKM2 O-GlcNAcylation to suppress PKM2 catalytic activity that increases aerobic glycolysis and tumor growth (Singh et al., 2020). The S-nitrosylation of PKM2 by JSD26 intraperitoneal injection protects against AKI in mice (Zhou et al., 2023). The modification at Cys358 by acrolein leads to PKM2 inactivation and aberrant glycolysis to promote renal fibrosis progression in high-fat diet-streptozotocin-induced diabetic kidney disease (DKD) mice. Treatment with acrolein scavengers (hydralazine and carnosine) obviously attenuates PKM2 activity and renal fibrosis (Kuo et al., 2023). Inhibiting neddylation modification by MLN4924 treatment induces tetramerization and activates PKM2 to increase glycolysis against cancer cell growth (Zhou et al., 2019). These data demonstrate that targeting post-translational modification of PKM2 is a promising therapeutic approach to treat renal and various diseases.

## 3 The role of PKM2 in kidney disease

### 3.1 Metabolism regulation

**FIGURE 3:**
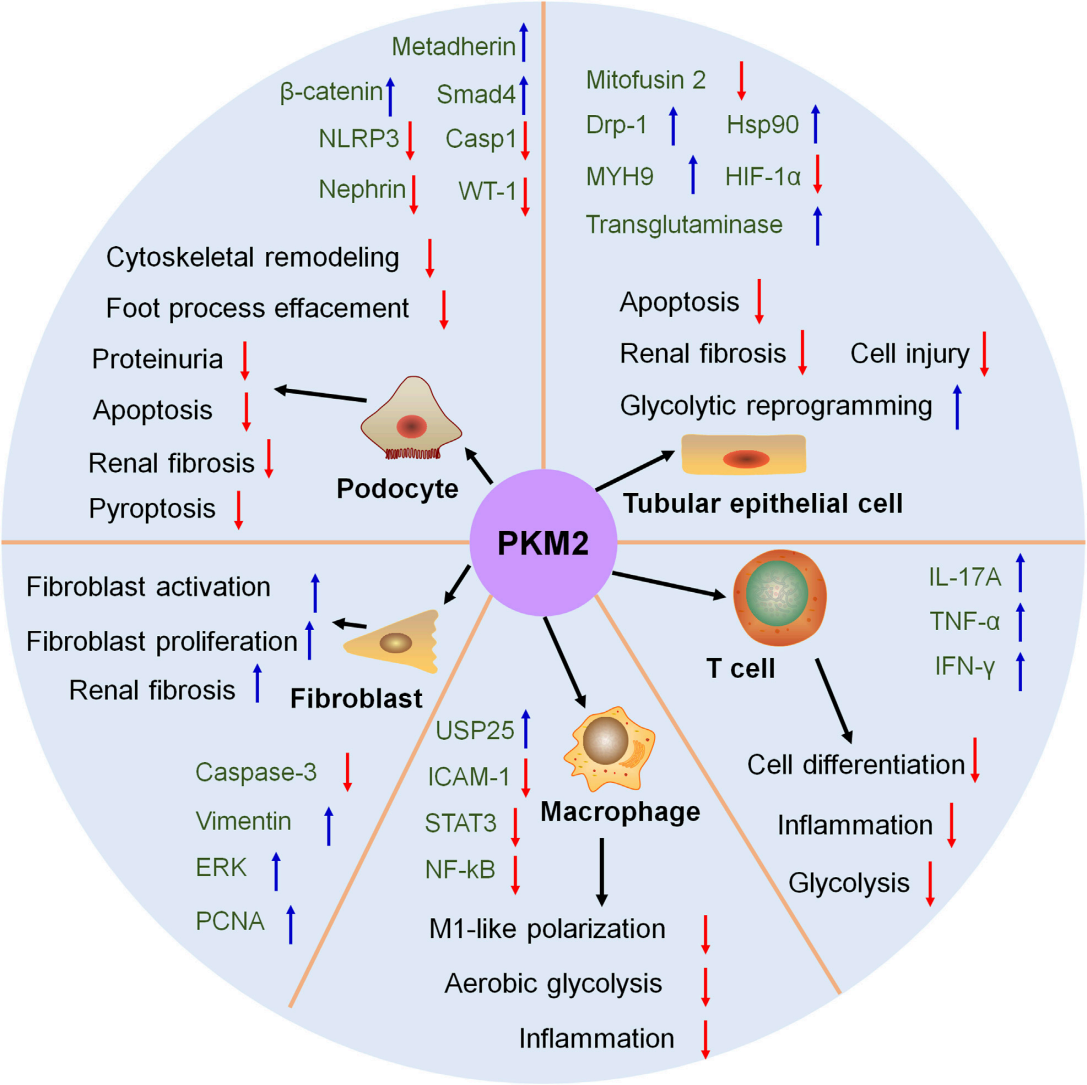
The role of PKM2 in kidney. In tubular epithelial cell, podocyte, fibroblast, macrophage and T cell, PKM2 participate in kidney disease and treatment. Drp-1, dynamin-related protein-1; ERK, extracellular regulated protein kinase; HIF-1α, hypoxia inducible factor-1; HSP90, heat shock protein 90; ICAM-1, intercellular cell adhesion molecule-1; IFN-γ, interferon gamma; IL-17A, interleukin-17A; MYH9, myosin heavy chain 9; NF-κB, nuclear factor kappa-B; NLPR3, NOD-like receptor thermal protein domain associated protein 3; PCNA, proliferating cell nuclear antigen; STAT3, signal transducer and activator of transcription 3; TNF-α, tumor necrosis factor-α; USP25, ubiquitin specific peptidase 25; WT-1, Wilm’s tumor-1.

PKM2 plays a vital role in regulating the glycolytic reprogramming in multiple renal cells and various kidney diseases (Figure 3). In tubular epithelial cell, the phosphorylation of PKM2 at Tyr105 promotes its dimer formation and translocation into the mitochondria after treatment with staurosporine or cisplatin in AKI. Hsp90 triggers the activation of PKM2-Akt signaling pathway to exhibit antiapoptotic effect against heat-stress injury in AKI (Chen et al., 2020). Mitochondrial PKM2 binds myosin heavy chain 9 to facilitate dynamin-related protein 1-mediated mitochondrial fragmentation, and the loss of PKM2 attenuates mitochondrial fragmentation and suppresses renal tubular injury and cell death by cisplatin (Xie et al., 2023). PKM2 interacts with mitofusin 2 to enhance mitochondrial fusion and oxidative phosphorylation, and suppress glycolysis (Li et al., 2019). Metabolomic study reveals that in cisplatin-induced normal kidney tubular epithelial NRK-52E cells, the excretion of PKM2 is significantly elevated in the media (Kim et al., 2022). PKM2 serves as the direct target of miR-144-5p. The long non-coding RNA (lncRNA) Opa-interacting protein 5 antisense RNA 1 targets miR-144-5p/PKM2 axis to attenuate the apoptosis of renal epithelial cells induced by cisplatin (Chang et al., 2022). Additionally, the activation of ERK modulated glycolysis and resulted in the reduced reserve respiratory capacity during cisplatin treatment (Suman et al., 2024).

PKM2 is significantly increased in glomeruli of patients with DKD and corelated with estimated glomerular filtration rate, which involves in preserving kidney function. The elevated PKM2 in circulation functions as biomarkers in DKD (Gordin et al., 2019). PKM2 protein is highly detected in the urine of DKD patients but not found in the urine of normal subjects. PKM2 is identified as the new biomarker for the early diagnosis of DKD (Park et al., 2023). DKD process is accompanied with Sirt3 suppression and PKM2 dimer formation. Sirt3 deficiency-induced abnormal glycolysis facilitates renal fibrosis (Srivastava et al., 2018). Sodium glucose cotransporter 2 inhibitor, empagliflozin, induces PKM2 dimer formation to normalize aberrant glycolysis, and eventually exhibits renal protection against DKD in proximal tubules (Li et al., 2020a). Besides, in the present of aminylation, tissue transglutaminase modulates glycolysis in normal and indoxyl sulfate-induced endothelial cell injury via activating PKM2 (Lin et al., 2023).

### 3.2 Podocyte injury

PKM2 is identified as a vital metabolic regulator for podocyte development (Figure 3). PKM2 is downregulated in podocytes from kidney biopsies of patients with DKD and hypertensive nephropathy (Luo et al., 2022; Chen et al., 2023). The injured glycolysis in podocytes enhances ornithine catabolism under diabetic conditions, and podocyte-specific loss of PKM2 aggravates ornithine catabolism to modulate cytoskeletal remodeling in podocytes in DKD (Luo et al., 2022). Podocyte-specific deletion of PKM2 in mice promotes angiotensin II-induced glomerular and podocyte injury with foot process effacement and proteinuria. Mechanistically, angiotensin II-induced glycolysis impairment aggravates an insufficient energy supply to the foot process and leads to podocyte injury (Chen et al., 2023). Podocyte-specific overexpression of PKM2 exhibits potent therapeutic effects on albumin/creatinine ratio, mesangial expansion, basement membrane thickness, and podocyte foot process effacement in streptozotocin-induced DKD mice (Fu et al., 2022). Podocyte-specific deletion of Smad4 alleviates DKD. Mechanistically, hyperglycaemia causes Smad4 localization to mitochondria in podocytes, and Smad4 directly binds to PKM2 and reduces the formation of active tetrameric form to decrease glycolysis (Li et al., 2020b).

In adriamycin-induced mice, podocyte-specific deletion of PKM2 exhibits limited energy metabolism that induces cell differentiation defects. Podocyte-specific knockout of PKM2 in mice worsens albuminuria and podocyte injury in adriamycin-induced mice (Yuan et al., 2020). Metadherin triggers the deaggregation of PKM2 tetramers and facilitates PKM2 monomers to enter the nucleus in podocyte to accelerate podocyte injury and proteinuria. Podocyte-specific knockout of metadherin attenuates proteinuria, podocyte injury and glomerulosclerosis after advanced oxidation protein products challenge or in adriamycin-induced mice (Chen et al., 2023).

In AKI, podocyte-specific deletion of PKM2 alleviates LPS-induced inflammation and apoptosis via the activation of β-catenin and the loss of Wilms' Tumor 1 and nephrin. These data reveals PKM2 as a promising therapeutic target for AKI (Alquraishi et al., 2022). The elevated PKM2 and increased glycolysis is detected during renal fibrosis. These contribute to hypoxic and acidic environment, and eventually suppress podocyte proliferation and differentiation and accelerate renal interstitial fibrosis. These results elicit that increased glycolysis-induced energy metabolism recodification influences podocyte number and function and aggravates fibrosis (Li et al., 2018).

PKM2 also participate in podocyte death. Podocyte-specific deletion of PKM2 contributes to glomerular and podocyte injury that involves in reduced glycolysis, cytoskeletal remodeling and podocyte apoptosis (Chen et al., 2023). The inhibition of dihydroxyacetone phosphate exhibits protective property on podocyte pyroptosis via downregulating PKM2 expression (Zhang et al., 2023).

### 3.3 Fibroblast activation and proliferation

Fibroblast-specific loss of PKM2 inhibits fibroblast proliferation and triggers tubular epithelial cell death during AKI process (Figure 3). PKM2-mediated fibroblast proliferation activates pro-survival signals to reduce tubular cell death (Ye et al., 2021). Overexpression of PKM2 triggers fibroblast activation and renal interstitial fibrosis that is accompanied by elevated glycometabolism, which elicits the important role of metabolic reprogramming in renal interstitial fibrosis (Yin et al., 2018). The knock-out of a multifunctional E3 ubiquitin-protein ligase, WWP2, facilitates myofibroblast proliferation and suppresses its activation. The loss of WWP2 sacrifices glycolysis and boosts mitochondrial respiration to activate fatty acid oxidation and the pentose phosphate pathway, which is the promising therapeutic target for renal fibrosis (Chen et al., 2024).

### 3.4 Macrophage polarization

Ubiquitin-specific protease 25 (USP25)-PKM2-aerobic glycolysis axis positively modulates M1-like polarization and accelerates ischemic AKI in mice, indicating potential therapeutic targets for AKI treatment (Figure 3). Mechanistically, USP25 controls aerobic glycolysis and lactate production during M1-like polarization via PKM2 (Yang et al., 2023). Besides, the regulation of PKM2 modulates inflammation by phosphorylating STAT3 and NF-κB in DKD that delays the differentiation of macrophages to M1 cells, and the downregulation of phosphorylated PKM2 is beneficial for DKD treatment (Li et al., 2020).

### 3.5 T cell regulation

Even few researches report the effect of PKM2 on T cell regulation in the kidney, the regulatory role of PKM2 on T cell regulation is confirmed in other tissues (Figure 3). PKM2 participates in T cell biology, and the upregulation, phosphorylation and nuclear accumulation of PKM2 is observed in CD4^+^ T cells. The activation of PKM2 by TEPP-46 treatment restricts T helper 17 and T helper 1 cells development and alleviates experimental autoimmune encephalomyelitis (Angiari et al., 2020). PRAK-Nrf2-mediated antioxidant signaling is a metabolic checkpoint that controls Th17 cell glycolysis and differentiation via restoring PKM2 phosphorylation to exhibit potent Th17 cell antitumor immunity (Zhao et al., 2023). Specific deletion of PKM2 in inflammatory hepatic CXCR3^+^ Th17 cells has ability to reverse inflammatory vigor and non-alcoholic fatty liver disease severity (Moreno-Fernandez et al., 2021). PKM2 is increased in infiltrated T lymphocytes of vascular lesions. Extracellular vesicles (EVs) from PKM2-activated T lymphocyte triggers abdominal aortic aneurysm progression through facilitating macrophage redox imbalance and migration (Dang et al., 2022). These data confirm the regulatory effect of PKM2 on T cell.

## 4 The activators and inhibitors of PKM2 in kidney disease

### 4.1 Activators

TEPP-46, a small-molecule PKM2 activator, leads to PKM2 tetramer formation through enhancing PKM2 subunit interactions and increasing PKM2 enzymatic activity (Table 2). Several studies reveal the protective role of TEPP-46 in DKD. The PKM2 activator TEPP-46 increases the glycolytic activity and lactate production in the kidney against DKD, suggesting targeting PKM2 as a promising therapeutic target for DKD treatment (Bertelsen et al., 2021). PKM2 activator TEPP-46 that promotes tetramerization enhances the interaction of endocytic trafficking through the versatile networks of Hsp70s and rewrites the crosstalk of EGFR signal transduction circuits and metabolic stress to promote resilience in DKD (Wang et al., 2022). PKM2 activation by TEPP-46 treatment improves tubular phenotype through suppressing the epithelial-mesenchymal transition program and normalizing aberrant glycolysis in high glucose-induced renal tubular epithelial cell and DKD model (Liu et al., 2021).

**TABLE 2:** The roles of PKM2 activators and inhibitors in kidney and other diseases.

Compound	Effects	Disease	Proposed function	References
Activators				
TEPP-46	Promoting PKM2 tetramer formation and increasing PKM2 enzymatic activity	DKD	Increasing the glycolytic activity and lactate production	Bertelsen et al. (2021)
		DKD	Promoting tetramerization enhances the interaction of endocytic trafficking	Wang et al. (2022b)
		DKD	Suppressing the epithelial-mesenchymal transition program and normalizing aberrant glycolysis	Liu et al. (2021)
DASA-58	Promoting PKM2 tetramerization and reducing lactate secretion	Liver fibrosis	Suppressing glycolysis and inflammation	Rao et al. (2022)
		Angiogenesis	Promoting glycolysis and mitochondrial fusion	Ren et al. (2020)
		Multiple sclerosis	Suppressing IL-17-producing T helper cell 17 development	Seki et al. (2020)
Mitapivat	Promoting PKM2 tetramer formation and increasing PKM2 enzymatic activity	PKD	Restoring activity of the red blood cell PK enzyme	Al-Samkari et al. (2022), Glenthøj et al. (2022)
		α-Thalassaemia and β-thalassaemia	Increasing haemoglobin concentration	Kuo et al. (2022)
TP-1454	Promoting PKM2 tetramer formation and increasing PKM2 enzymatic activity	Advanced solid tumors	Modulating cellular metabolism and enhancing response to checkpoint inhibitors	Weagel et al. (2022)
Celastrol	Altering the spatial conformation to reduce the enzyme activity	Non-alcoholic fatty liver disease	Suppressing lipid accumulation, inflammation and fibrosis	Fan et al. (2022)
Modified Hu-lu-ba-wan	Increasing PKM2 protein expression	DKD	Facilitateing mitochondrial dynamic homeostasis	Gong et al. (2023)
Inhibitors				
Shikonin	Decreasing PKM2 enzymatic activity	AKI	Decreasing the histopathological symptoms and apoptosis	Wu et al. (2021)
		AKI	Inhibiting renal oxidative stress, inflammatory and tubular epithelial cell apoptosis	Peng et al. (2022)
		Unilateral ureteral obstruction	Inhibiting renal fibrosis	Wei et al. (2019)
		NRK-52E cells	Modulating mitochondrial membrane potential	Tong et al. (2018)
		DKD	Inhibiting renal oxidative stress and inflammation	Balaha et al. (2023), Zhu et al. (2023)
		Liver fibrosis	Modulating PKM2-mediated aerobic glycolysis	Zheng et al. (2020)
Compound 3k	Triggering PKM2 tetramer disruption	Idiopathic pulmonary fibrosis	Stabilizing TGF-β1 receptor I and enhancing TGF-β1 signaling	Gao et al. (2022)
		Ovarian cancer	Inhibiting glycolysis and reprograms metabolism	Park et al. (2021)
Compound 3h		Prostate cancer	Attenuating apoptotic and autophagic cell death	Jiang et al. (2022)
Microcystin-RR	Inhibiting PKM2 expression and phosphorylation	Unilateral ureteral obstruction	Restoring the inhibited expression of MMP-7 and MMP-13, and reducing the upregulated expression of MMP-9	Ren et al. (2022)
*Dendropanax morbifera*	Reducing PKM2 expression	DKD	Suppressing oxidative stress and inflammation	Sachan et al. (2020)
Huangqi-Danshen decoction	Reducing PKM2 expression	Adenine-induced CKD	Reshaping glucose metabolism profiles	Huang et al. (2023)
Qian Yang Yu Yin granule	Reducing PKM2 expression	Hypertensive nephropathy	Modulating metabolic reprogramming	Qian et al. (2021)
Tianshu capsule	Reducing serum PKM2 expression	Spontaneous hypertension	Normalizing energy metabolism	Gao et al. (2019)
LncRNA ARAP1	Promoting tetrameric PKM2 formation	DKD	Normalizing aberrant glycolysis and fibrosis	Li et al. (2023a)

AKI, acute kidney injury; CKD, chronic kidney disease; DKD, diabetic kidney disease.

DASA-58 is able to induce PKM2 activation by promoting PKM2 tetramerization and reducing lactate secretion (Table 2). Pharmacological activation of PKM2 by DASA-58 partially counters glycolysis and inflammation during liver fibrosis process (Rao et al., 2022). DASA-58 treatment facilitates the proliferation of vascular resident endothelial progenitor cell and enhances PKM2 activity via promoting glycolysis and mitochondrial fusion (Ren et al., 2020). Besides, treatment with TEPP-46 or DASA-58 obviously hinders IL-17-producing T helper cell 17 development against multiple sclerosis (Seki et al., 2020). Even if few studies report the effect of DASA-58 in kidney disease, the above-mentioned data provide a promising prospect for DASA-58 in kidney disease treatment.

Mitapivat, also called AG-348, is the first-line oral small molecule allosteric activator of PK (Table 2). Several clinical trials have confirmed the beneficial role of mitapivat in pyruvate kinase deficiency (PKD) treatment, and mitapivat has been granted orphan drug designation by the FDA for PKD. A phase 3, randomized, placebo-controlled clinical trial demonstrates that mitapivat substantially elevates hemoglobin level, reduces hemolysis, and improves patient-reported outcomes in patients with PKD (Al-Samkari et al., 2022). Another multicentre, open-label, single-arm, phase 3 trial shows that mitapivat functions as a novel therapy and becomes first disease-modifying agent for PKD that alleviates transfusion burden in adult patients who accepts regular transfusions (Glenthøj et al., 2022). Besides, an open-label, multicentre, phase 2 study reveals that mitapivat also exhibits potent therapeutic effect against α-thalassaemia and β-thalassaemia, and haemoglobin concentration significantly increases after treatment (Kuo et al., 2022).

TP-1454, derived from SGI-9380, enhances the bind of two dimer PKM2 as a glue of monomer PKM2 (Xu et al., 2014). TP-1454 is a potent PKM2 activator and has enter clinical trials as the first oral PKM2 activator for advanced solid tumors treatment (Weagel et al., 2022). In addition, PKM2 is identified as a major celastrol-bound protein, and celastrol binds to the residue Cys31 and further alters the spatial conformation to reduce the enzyme activity of PKM2 in hepatic macrophages against non-alcoholic fatty liver disease (Fan et al., 2022).

PKM2 is also the therapeutic target of traditional medicine against kidney disease. Modified Hu-lu-ba-wan exhibits beneficial effect on DKD patients and animal model through facilitating mitochondrial dynamic homeostasis. The one of its components, berberine, has a high affinity with PKM2, and prevent apoptosis by enhancing PKM2-mediated mitochondrial dynamic homeostasis (Gong et al., 2023).

### 4.2 Inhibitors

Shikonin and alkannin, enantiomeric pair of pigments derived from *Lithospermum erythrorhizon* roots, are identified as potent and highly selective PKM2 inhibitor (Table 2). The protective role of shikonin has been confirmed in kidney diseases (Wei et al., 2019; Wu et al., 2021; Peng et al., 2022). The inhibition of PKM2 by shikonin notably suppresses the expression of HIF-1α and apoptosis-related factors such as BNIP3, Bax, and caspase-3, while the inhibition of PKM2 by shikonin significantly improves the histopathological symptoms of LPS-induced AKI. The inhibition of PKM2 by shikonin significantly ameliorates the histopathological symptoms in LPS-induced AKI mice and inhibits apoptosis via downregulating BNIP3, Bax, and caspase-3 (Wu et al., 2021). Shikonin inhibits renal oxidative stress, inflammatory and tubular epithelial cell apoptosis through regulating NOX4/PTEN/AKT pathway against sepsis-induced AKI rat model (Peng et al., 2022). The blockade of glycolysis by shikonin obviously attenuates renal fibrosis through modulating PKM2 in unilateral ureteral obstruction mice (Wei et al., 2019). Shikonin effectively controls mitochondrial membrane potential to exhibit renal protection in high glucose-induced NRK-52E cells (Tong et al., 2018). Shikonin suppresses DKD progression and gentamicin-induced renal injury through inhibiting renal oxidative stress and inflammation (Balaha et al., 2023; Zhu et al., 2023). In addition, PKM2 antagonist (shikonin) and its allosteric agent (TEPP-46) substantially attenuates liver fibrosis by modulating PKM2-mediated aerobic glycolysis (Zheng et al., 2020). As for alkannin, few studies investigate its renal protection, but alkannin exhibits potent therapeutic property on Alzheimer’s disease and anti-cancer treatment (Chang et al., 2020; Hosoi et al., 2023).

The selectivity of compound 3k is higher than shikonin for PKM2 (Gao et al., 2022). Compound 3k triggers PKM2 tetramer disruption to delay fibrosis progression in idiopathic pulmonary fibrosis (Gao et al., 2022). Treatment with compound 3k hinders glycolysis and reprograms metabolism against ovarian cancer (Park et al., 2021). Besides, comparing with compound 3k, compound 3h has a higher affinity with PKM2. Treatment with compound 3h hinders glycolytic pathways to attenuate apoptotic and autophagic cell death against prostate cancer cells (Jiang et al., 2022).

PKM2 severs as a potential therapeutic target for natural product and antisense against kidney diseases. Microcystin-RR directly binds to PKM2 and suppresses phosphorylated PKM2 at Lys105 to impair PKM2-HIF-1α pathway against chronic kidney disease (CKD) (Ren et al., 2022). The aquatic extract of *Dendropanax morbifera* alleviates PKM2 expression against DKD and renal fibrosis via ameliorating oxidative stress and inflammation (Sachan et al., 2020). Huangqi-Danshen decoction, a Chinese herbal preparation, attenuates PKM2 expression to reshape glucose metabolism profiles against adenine-induced CKD (Huang et al., 2023). Qian Yang Yu Yin granule suppresses PKM2 expression in hypertensive nephropathy rat model and modulates metabolic reprogramming via HIF-1α/PKM2 positive feedback loop (Qian et al., 2021). Tianshu capsule alleviates serum PKM2 expression to normalize energy metabolism in spontaneously hypertensive rat model (Gao et al., 2019). Besides, natural antisense lncRNA ARAP1 knock-down suppresses dimeric PKM2 expression and restores tetrameric PKM2 formation to normalize aberrant glycolysis and fibrosis in DKD models (Li et al., 2023).

## 5 Conclusion and perspectives

Glycolysis is the key energy resource for kidney, and PKM2 plays a vital role in glycolysis. PKM2 is not only the rate-limiting enzyme that mediates PEP to pyruvate, also functions as a co-transcription factor that triggers the upregulation of glycolysis-related genes including MYC, SLC2A1, LDHA and PDK1 (Li et al., 2023). PKM2 monomer has 531 amino acids and owns four domains (N, A, B and C). The inactive monomer, nearly inactive dimer, inactive T state tetramer, and active R state tetramer are four states of PKM2 protein. The post-translational modification of PKM2 modulates its enzymatic activity and nuclear localization. PKM2 phosphorylation at Tyr105 and Ser37 inhibits active tetramer formation and induces nuclear translocation, and the acetylation of PKM2 at Lys433, Lys305 and Lys66 also influences its enzymatic activity and nuclear translocation. The SUMOylation at Lys270 triggers PKM2 dimeric formation and nuclear translocation, while the SUMOylation at Lys336 enhances PKM2 enzymatic activity. The deubiquitination of PKM2 at Ser57 by HAUSP protects PKM2 from degradation. Besides, the lactylation and the modification at Cys358 by acrolein also affects PKM2 enzymatic activity to regulate glycolysis. These data indicates that the post-translational modification of PKM2 is responsible for PKM2 enzymatic activity and nuclear translocation that is the promising therapeutic target.

Emerging evidences show that the aberrant glycolysis contributes to kidney disease progression accompanying PKM2 dysfunction. PKM2 participates in kidney disease and treatment through modulating metabolism regulation, podocyte injury, fibroblast activation and proliferation, macrophage polarization, and T cell regulation. Notably, the expression and enzymatic activity of PKM2 is substantially disturbed in kidney and targeting PKM2 has been proved a promising therapeutic target, especially in DKD and AKI that is consistent with previous study (Rabelink and Carmeliet, 2018). Notably, DKD and AKI accompany with the accumulation of glycolytic intermediate products (Rabelink and Carmeliet, 2018). The obvious elevation of PKM2 is observed in glomeruli of patients with DKD (Gordin et al., 2019), and the knock-down of PKM2 in podocyte aggravates glomerular injury and albuminuria in DKD mice (Qi et al., 2017). In AKI, PKM2 severs as a novel biomarker for early detection (Cheon et al., 2016), and the deletion of PKM2 in several kidney cells is beneficial in AKI. The deletion of PKM2 in podocyte attenuates AKI, and the deletion of PKM2 in fibroblast triggers fibroblast activation and AKI progression. Besides, PKM2 also controls macrophage polarization to participate in AKI. The possible reason that PKM2 is more important in DKD and AKI than other kidney diseases is that DKD and AKI suffer aberrant glycolysis and energy metabolism more during their progression. These results reveal that targeting PKM2 is emerging as a promising therapeutic approach for DKD and AKI treatment.

Interestingly, both PKM2 activator TEPP-46 and PKM2 inhibitor shikonin exhibits protective effect on kidney diseases, suggesting the importance of PKM2 during kidney disease progression. Importantly, these results remind us that the deep investigation of PKM2 pleiotropic effects are urgent before the application in clinical. The novel positron emission tomography [^18^F]DASA-23 helps to determine PKM2 level in human to visualize intracranial malignancies that is a useful tool to explore of PKM2 pleiotropic effects in kidney (Beinat et al., 2020). Both the regulation of PKM2 post-translational modification and pharmacological modulation of PKM2 is the promising approach to control PKM2 activity and kidney disease progression. PKM2 activator (TEPP-46, DASA-58, mitapivat and TP-1454) and PKM2 inhibitor (shikonin, alkannin, compound 3k and compound 3h) have exhibited potential therapeutic property in kidney disease, which provides candidates for kidney disease in further pre-clinical and clinical investigation. Another limitation that hinders PKM2 as a therapeutic target is restricted clinical trials in kidney disease and other disease. Clinical trials demonstrates that PKM2 is the potential biomarker in inflammatory bowel disease and Crohn’s disease (Almousa et al., 2018), and elevated plasma PKM2 is associated with poorer prognosis of pancreatic and peri-ampullary cancer in clinical (Bandara et al., 2018). The increased PKM2 expression are more frequent in cirrhotic liver than non-cirrhotic liver, and is associated with poor survival rates in both cirrhotic liver and non-cirrhotic liver (Liu et al., 2017). Although the clinical and related renal studies referring PKM2 are limited, the promising potential of PKM2 has been verified, which provides the candidates for kidney disease treatment.

Overall, PKM2 is the promising therapeutic target for kidney disease. The deep investigation of PKM2 pleiotropic effects in kidney and clinical trial are urgently needed to verify the therapeutic effect of PKM2 activator/inhibitor to benefit patients.
